# Aldose reductase from *Schistosoma japonicum*: crystallization and structure-based inhibitor screening for discovering antischistosomal lead compounds

**DOI:** 10.1186/1756-3305-6-162

**Published:** 2013-06-05

**Authors:** Jian Liu, David H Dyer, Jingdong Cheng, Jipeng Wang, Shuqi Wang, Zhong Yang, Xiaoning Wang, Wei Hu

**Affiliations:** 1 Key Laboratory of Parasite and Vector Biology of MOH, Institute of Parasitic Diseases, Chinese Center for Disease Control and Prevention, 207 Rui-Jin Road II, Shanghai 200025, China; 2 Department of Microbiology and Microbial Engineering, School of Life Sciences, Fudan University, 220 Han-Dan Road, Shanghai 200433, China; 3 Department of Entomology, University of Wisconsin-Madison, 1630 Linden Drive, Madison, WI 53706, USA; 4 Institutes of Biomedical Sciences, Fudan University, 130 Dong-An Road, Shanghai 200032, China

**Keywords:** *Schistosoma japonicum*, Aldose reductase (AR), Structure, Virtual screening, Drug target

## Abstract

**Background:**

Schistosomiasis is a neglected tropical disease with high morbidity and mortality in the world. Currently, the treatment of this disease depends almost exclusively on praziquantel (PZQ); however, the emergence of drug resistance to PZQ in schistosomes makes the development of novel drugs an urgent task. Aldose reductase (AR), an important component that may be involved in the schistosome antioxidant defense system, is predicted as a potential drug target.

**Methods:**

The tertiary structure of *Schistosoma japonicum* AR (*Sj*AR) was obtained through X-ray diffraction method and then its potential inhibitors were identified from the Maybridge HitFinder library by virtual screening based on this structural model. The effects of these identified compounds on cultured adult worms were evaluated by observing mobility, morphological changes and mortality. To verify that *Sj*AR was indeed the target of these identified compounds, their effects on recombinant *Sj*AR (r*Sj*AR) enzymatic activity were assessed. The cytotoxicity analysis was performed with three types of human cell lines using a Cell Counting Kit-8.

**Results:**

We firstly resolved the *Sj*AR structure and identified 10 potential inhibitors based on this structural model. Further *in vitro* experiments showed that one of the compounds, renamed as AR9, exhibited significant inhibition in the activity of cultured worms as well as inhibition of enzymatic activity of r*Sj*AR protein. Cytotoxicity analysis revealed that AR9 had relatively low toxicity towards host cells.

**Conclusions:**

The work presented here bridges the gap between virtual screening and experimental validation, providing an effective and economical strategy for the development of new anti-parasitic drugs. Additionally, this study also found that AR9 may become a new potential lead compound for developing novel antischistosomal drugs against parasite AR.

## Background

Schistosomiasis is a major tropical disease in developing countries. It is estimated that over 200 million people from 76 countries and territories are suffering from this disease [1]. The disease is usually caused by one of three schistosome species: *Schistosoma japonicum*, *Schistosoma mansoni* and *Schistosoma haematobium*. In China, *S. japonicum* is the primary pathogen of this disease [2]. Currently, the treatment of schistosomiasis depends almost exclusively on praziquantel (PZQ), and this drug has been widely used for nearly 40 years because of its high efficacy but low cost [3]. However, the long-term utilization of one drug can result in drug-resistant parasites. Decreased susceptibility of *S. mansoni* and *S. haematobium* to PZQ has already been identified in previous studies [4,5]. Although no reduced susceptibility of *S. japonicum* has been proven to date, the efficacy of this drug is found to vary in different strains within this species [6]. Therefore there is an urgent need to develop novel antischistosomal lead compounds, and the identification of ideal drug targets is an important step toward this goal.

Antioxidant defense is an essential mechanism for schistosomes to cope with damage from host immune- and self-generated reactive oxygen species (ROS) [7]. Many redox-associated proteins such as thioredoxin glutathione reductase (TGR), peroxiredoxin (Prx) and thioredoxin (Trx) have been demonstrated to be involved in this system in previous studies [8-11]. Most of these proteins are considered as potential drug targets, as one example, two recently discovered prospective antischistosomal compounds, auranofin and oxadiazoles, were developed with TGR as drug target [9,12]. Although no research has shown that *S. japonicum* AR participates in the antioxidant pathway, in other organisms, AR is believed to be an important antioxidant component. Spycher *et al.*[13] found that the levels of AR mRNA were up-regulated under oxidative stress in rat smooth muscle cells. Furthermore, the levels of AR expression as well as its activity were increased during hyperglycemia and other oxidative stress-induced diseases in humans [14,15]. Additionally, many byproducts of oxidative stress, such as methylglyoxal and 3-deoxyglucosone, have been shown to be excellent substrates of AR [16]. Considering both these conclusions and the antioxidant requirement of schistosomes, it is reasonable to speculate that *Sj*AR might also participate in the antioxidant pathway and protect the worms from host ROS attack. In addition to the above, AR has also been demonstrated to play an important role in aldehyde detoxification, steroid metabolism, energy supply, cellular proliferation, apoptosis and senescence [17-20]. Its multiple functions suggest that it may represent a key enzyme in schistosomes.

In the present study, we successfully resolved the tertiary structure of recombinant *Sj*AR and identified 10 inhibitor candidates through molecular docking based on the obtained structural model. We then assessed the activity inhibition of these compounds on cultured worms. To further confirm that the *Sj*AR protein was indeed the target of the selected compounds, we investigated the effect of the identified compounds on the enzymatic activity of recombinant *Sj*AR (r*Sj*AR). The cytotoxicity of the active compounds towards the host cell was evaluated as well. Finally, one compound, renamed as AR9, was determined to effectively inhibit the activity of cultured worms but show relatively low cytotoxicity against host cells, which suggests its potential use as a lead compound from the selected candidate inhibitors. The work presented here bridges the gap between virtual screening and experimental validation, providing an effective and economical strategy to develop novel anti-parasitic drugs.

## Methods

### Materials

Protein crystallization kits were purchased from Hampton Research Corporation (USA). NADPH was obtained from Roche (Switzerland). DL-glyceraldehyde and PZQ came from Sigma (USA). Small molecules identified by virtual screening were purchased from Maybridge HitFinder library (USA). RPMI 1640, DMEM and bovine serum (Newborn calf serum and fetal bovine serum) came from Invitrogen (USA). The recombinant *Sj*AR-pET28a plasmid was constructed previously and stored in our laboratory. BL21 (DE3) and Hep G2 cells were also stored in our laboratory. 293T and HeLa cell lines were kindly provided by Hongyan Wang (School of Life Sciences, Fudan University, China). *S. japonicum* cercaria was provided by the pathogen biology laboratory of the National Institute of Parasitic Diseases, Chinese Center for Diseases Control and Prevention. Specific pathogen-free Kunming female mice were purchased from the Shanghai Experimental Animal Center, Chinese Academy of Sciences (China).

### Expression and purification of r*Sj*AR

The recombinant plasmid *Sj*AR-pET28a was transformed into *E. coli* BL21 (DE3) cells and cultured in Luria-Bertani (LB) medium plus 50 μg/ml kanamycin. Isopropylthio-β-D-galactoside (IPTG), 1 mM, was added to the medium to induce protein expression, and then the cells were cultured for an additional 6 h. The cells were harvested by centrifugation, and pellets were resuspended in lysis buffer (20 mM Tris–HCl, 500 mM NaCl, 1 mM PMSF, pH 8.0). Subsequently, the cells were disrupted by ultrasonic waves for 5 min in 2 s pulses at 160 W. The whole cell lysate was clarified by centrifugation at 10,000 × *g* for 30 min at 4°C. The resulting supernatant was purified sequentially using immobilized metal ion affinity chromatography, anion-exchange chromatography, and finally, size-exclusion chromatography. The purified protein was stored in 20 mM Tris–HCl (pH 6.2), 100 mM NaCl, 5 mM DTT. The r*Sj*AR protein was concentrated by ultrafiltration using Millipore Ultrafiltration System with a molecular weight cut off at 10 KDa. Protein concentration was determined by a Bradford Protein Assay Kit (Glory, USA).

### Crystallization

Initial crystallization conditions were screened in Tissue Culture Test Plates 24 (TPP) by the hanging-drop method at 291 K, using the sparse-matrix method [21] implemented in the Crystallization Screens Kits (included Index, Crystal Screen, Crystal Screen 2, PEG/Ion Screen and PEG/Ion 2 Screen and SaltRx) from Hampton Research. Three protein concentrations were adopted: 24 mg/ml, 12 mg/ml and 6 mg/ml. A total of 1 μl protein solution was mixed with 1 μl well solution and equilibrated against 200 μl reservoir solution. Crystallization leads were identified in over 10 of these conditions. One initial condition (PEG/Ion Screen: 0.2 M Sodium fluoride, 20% w/v Polyethylene glycol 3,350, pH 7.3), which produced single crystals, was optimized to obtain crystals suitable for diffraction analysis. The final optimal conditions were 12 mg/ml protein and a reservoir solution consisting of 0.2 M Sodium fluoride, 30% w/v, Polyethylene glycol 3,350 (pH 7.1).

### Data collection and processing

Crystals were flash-cooled in liquid nitrogen with a cryoprotectant containing only reservoir solution. Diffraction data were collected at Beamline BL17U at the Shanghai Synchrotron Radiation Facility and processed with the package HKL-2000 using routine procedures [22]. The initial phases were calculated in the program PHASER [23], from the CCP4 suites, using a structure known *H. sapiens* AR (*Hsa*AR, PDB ID: 1ZUA) as a search model. The final model was manually built with COOT [24]. All computational refinements were performed using the refinement module phenix.refine of the PHENIX package [25]. The model quality was checked with the PROCHECK program, which showed good stereochemistry according to the Ramachandran plot for the structure.

### Molecular docking

To identify potential inhibitors of *Sj*AR, the Maybridge HitFinder library, which contains approximately 80,000 compounds, was chosen for *in-silico* screening with the model of the r*Sj*AR protein. Molecular docking was firstly performed with Sybyl v8.0 Surflex-Dock (http://www.tripos.com) followed by docking the top 5% hits with AutoDock 4.2 (http://autodock.scripps.edu/). The top 100 scoring compounds were selected out and exported to an Excel spreadsheet. To increase the selectivity of these compounds, they were also docked into the *Hsa*AR protein. The final obtained compounds conformed to the following principles: 1) the *Sj*AR protein-compound binding free energy was lower than −9 kCal/mol; and 2) the compound showed a greater binding preference to the *Sj*AR protein than to *Hsa*AR (the difference of binding free energy was higher than 2 kCal/mol).

### Inhibition studies on cultured worms

Mice infected with 80–100 cercariae were killed at 35 days-post-infection for worm collection. *S. japonicum* adult worms were obtained by perfusion and washed three times with sterile saline. Next, the worms were transferred to RPMI 1640 medium containing 300 μg/ml penicillin, 300 μg/ml streptomycin, 0.25 μg/ml amphotericin and 20% fetal bovine serum and then cultured for 2 h to make the worms discharge their gut contents. Two pairs of worms with good activity were selected and transferred to each well of a 24-well plate containing 2 ml of the preceding culture medium. Stocking solutions of compounds were prepared by dissolving 2 mg of the compounds in 0.4 ml dimethyl sulfoxide (DMSO) and then were added to a series of final concentrations (for initial screening, three concentrations of 5 μg/ml, 25 μg/ml and 50 μg/ml were assessed, while for the second screening, five concentrations of 1.25 μg/ml, 2.5 μg/ml, 5 μg/ml, 10 μg/ml, and 20 μg/ml were assessed). The worms in the control group were treated with equal amounts of the compound carrier. A PZQ treated group was also observed as a positive control. The test was repeated three times, and for each experimental condition, 12 worms in 3 wells were tested.

The worms were cultured at 37°C in an incubator with 5% CO_2_. The worm mobility, morphological changes and mortality were observed under an inverted microscope at 2 h, 24 h, 48 h and 72 h. Parasite death was defined as non-detectable activity in 2-minutes, accompanied by morphological and tegumental alterations [26]. The median lethal concentration (LC50) values for the identified active compounds were calculated by the software SPSS 18.0, with a confidence interval of 95%.

### Effect of compound AR9 on r*Sj*AR enzymatic activity

The enzymatic assay was described previously [27]. Briefly, the reaction was performed in 120 mM PBS buffer (pH 6.2), containing 1.5 mM DL-glyceraldehyde, 0.15 mM NADPH and 0.15 μM r*Sj*AR in a final volume of 200 μl. The mixture was first incubated at 37°C for 5 min, and then the reaction was initiated by adding the substrate of NADPH. For inhibition analysis, AR9 was added to the same reaction system to final concentrations of 5 μg/ml, 10 μg/ml, 20 μg/ml and 40 μg/ml. The reaction process was determined by monitoring the absorbance reduction at 340 nm due to the depletion of NADPH using a Model 680 Microplate Reader (Bio-Rad, USA).

### Scanning electron microscopy (SEM)

Schistosome samples were fixed with 2.5% glutaraldehyde in PBS buffer for 2 h and were then washed thoroughly three times with PBS buffer. The samples were fixed again in 1% osmium tetroxide in PBS buffer. After ethanol dehydration and critical point drying, they were mounted on microscope stubs, followed by gold sputtering for 3 min in an IB-3 ion-sputtering instrument. The SEM scanning was performed on an S-520 SEM (Hitachi, Japan) instrument with an accelerating voltage of 20 kV [28].

### Cytotoxicity assay

Cytotoxicity analysis was performed with three types of human cell lines (Hep G2, 293T and Hela cell lines) using a Cell Counting Kit-8 according to the protocol provided by the manufacturers (Beyotime, China). Briefly, the cells were cultured in a 96-well plate containing 100 μl DMEM medium (containing 10% fetal bovine serum) at a density of 5,000 cells per well at 37°C in 5% CO_2_. The cells were allowed to recover for 24 h and then exposed to various concentrations of compound or compound-carrier. When the cells in the negative control group (carrier alone) covered more than 90% of the surface of the well, 10 μl of WST-8 chromogenic agent was added to each well and then continuously incubated for 30 min. The absorbance at 450 nm was determined by using a Model 680 Microplate Reader (Bio-Rad, USA).

### Ethical approval

The animal work was approved by the Ethics Committee of the National Institute of Parasitic Diseases, Chinese Center for Disease Control and Prevention in Shanghai, China (Ref No: 20100525–1). Animal care and all procedures involving animals were performed in strict accordance with the Guidelines for the Care and Use of Laboratory Animals of the Ministry of Science and Technology of People’s Republic of China ([2006]398). All efforts were made to minimize suffering.

## Results

### Structure determination and description

The r*Sj*AR protein crystals diffracted to a resolution of 2.2 Å and belonged to the space group P2_1_2_1_2 with the unit cell parameters a = 67.49, b = 91.00 and c = 54.67 Å. Residues 211–221 were disordered and were not built into the structure model. After structure solution and computational refinement, the final model of *Sj*AR, validated using PROCHECK, had 92.5% of residues in the most favored regions of the Ramachandran plot and 7.5% of residues in the additionally allowed regions. The structure had Root Mean Square Deviation (RMSD) from ideality for bond lengths of 0.007 Å and for an angle of 1.023°. Further data collection and computational refinement statistics are summarized in Table 1.

**Table 1:** X-ray data-collection and structure refinement statistics

**Data collection and processing**	
Processing software	HKL2000
Synchrotron, beamline	SSRF, BL17U
Wavelength (Å)	0.9791
Space group	P2_1_2_1_2
Unit cell parameters a, b, c (Å, °)	a = 67.49, b = 91.00, c = 54.67
Resolution range (Å)^1^	30.0-2.20 (2.28-2.20)
Unique reflections	17617
Redundancy	6.4 (6.5)
Completeness (%)	99.8 (99.9)
R_merge_ (%)	12.5 (37.1)
I/σ(*I*)	13.8 (5.0)
**Refinement statistics**	
Refinement software	PHENIX
R_work_ (%)/R_free_(%)^2^	17.7/21.7
Average B factor, (Å^2^)	17.9
RMSD from ideal geometry, bonds (Å)	0.007
RMSD from ideal geometry, angles (°)	1.023
Protein atoms	2452
Water molecules	225
**Residues in the Ramachandran plot**	
Most favored region (%)	92.5
Allowed region (%)	7.5
Generously allowed region (%)	0
Disallowed region (%)	0

^1^Values in parentheses are for the highest resolution shell.

^2^*R*_free_ = ∑_Test_||*F*_obs_| –|*F*_calc_||/∑_Test_ |*F*_obs_|, where “Test” is a test set of about 5% of the total reflections randomly chosen and set aside before refinement.

**Figure 1:**
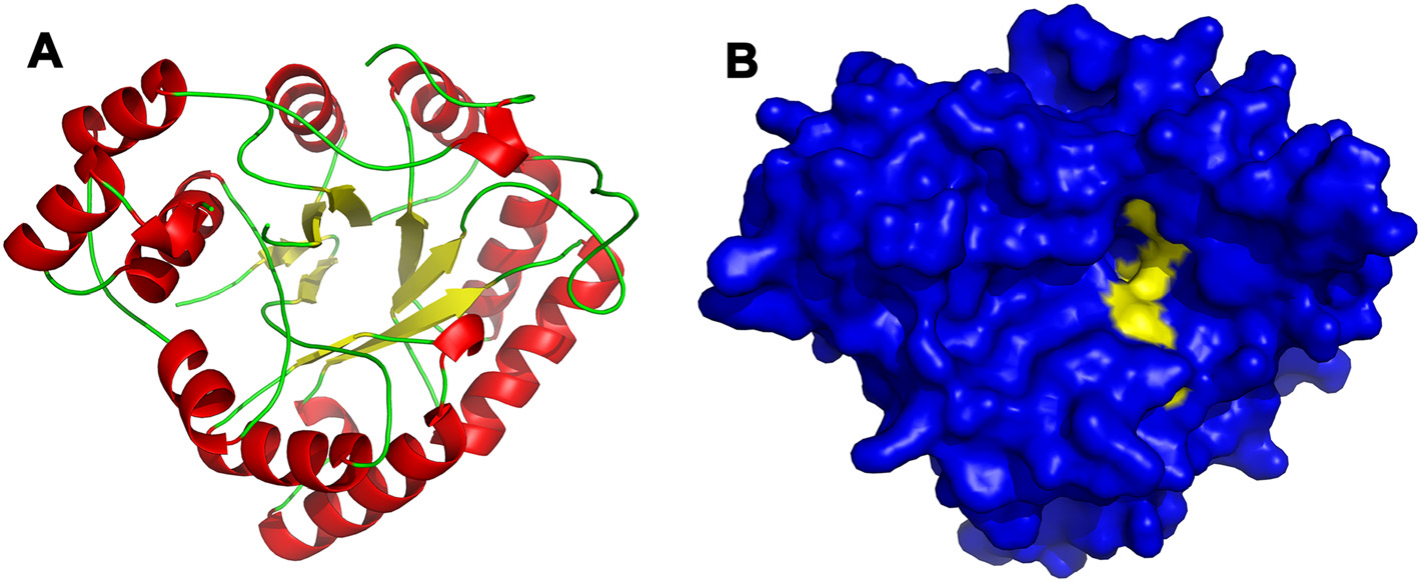
**Structure and predicted cofactor binding area of the *Sj*AR protein. A.** Cartoon diagram of *Sj*AR with α-helices colored red, β-sheets colored yellow and loops colored green. **B.** The structure of *Sj*AR is shown in a surface representation with the regions predicted to be involved in cofactor binding (by homology with *H. sapiens* AR) colored yellow.

The overall structure of *Sj*AR (PDB ID: 4HBK) showed an (α/β)_8_ barrel topology, which is the typical characteristic of the aldo-keto reductase superfamily (Figure 1A) [29,30]. By comparing the amino acid sequence with the homologous *Hsa*AR protein, we found both the residues at the substrate-binding site (Asp^43^, Tyr^48^, Lys^77^ and His^110^) and at the predicted inhibitor-binding sites (Tyr^48^, His^110^ and Trp^111^) were conserved [30,31]. These residues were located in the interior of the β-barrel, which provided a target area to begin docking (Figure 1B).

### Structure-based virtual screening

Although the *Sj*AR protein shares only 51% sequence identity with *Hsa*AR (PDB ID: 1ZUA), their tertiary structures are highly similar (RMSD: 0.865 Å). The AR protein family has highly conserved substrate binding sites, and all the nearby residues surrounding the binding sites are the same, which suggests that the region would be somewhat unsuitable for docking. Therefore, we compared the structural divergence near the potential inhibitor binding regions. Compared with the known *Hsa*AR-tolrestat complex structure [30], in the *Sj*AR structure, Lys^257^ penetrated into the binding regions, while Gln^297^ and Trp^20^ were moved away. Additionally, Phe^293^ replaced Cys^299^ to participate in the formation of the hydrophobic pocket. However, all of these residues had the potential to interact with inhibitors. The finally obtained 10 candidate compounds as well as their binding free energy with *Sj*AR and *Has*AR were listed in Table 2.

**Table 2:** The finally obtained 10 candidate compounds analyzed with AutoDock 4.2

**Compound ID**	**Structure**	**Binding free energy (kCal/mol)**		***Sj*AR-*Hsa*AR (kCal/mol)**
		***Sj*AR**	***Hsa*AR**	
AR1		−13.56	−11.42	2.14
AR2		−13.80	−10.61	3.19
AR3		−12.77	−10.47	2.30
AR4		−10.09	−6.09	4.00
AR5		−11.59	−9.52	2.07
AR6		−9.31	−7.00	2.31
AR7		−9.05	−6.34	2.71
AR8		−13.47	−10.99	2.48
AR9		−10.08	−8.07	2.01
AR10		−9.15	−3.78	5.37

### Inhibition studies on cultured worms

**Figure 2:**
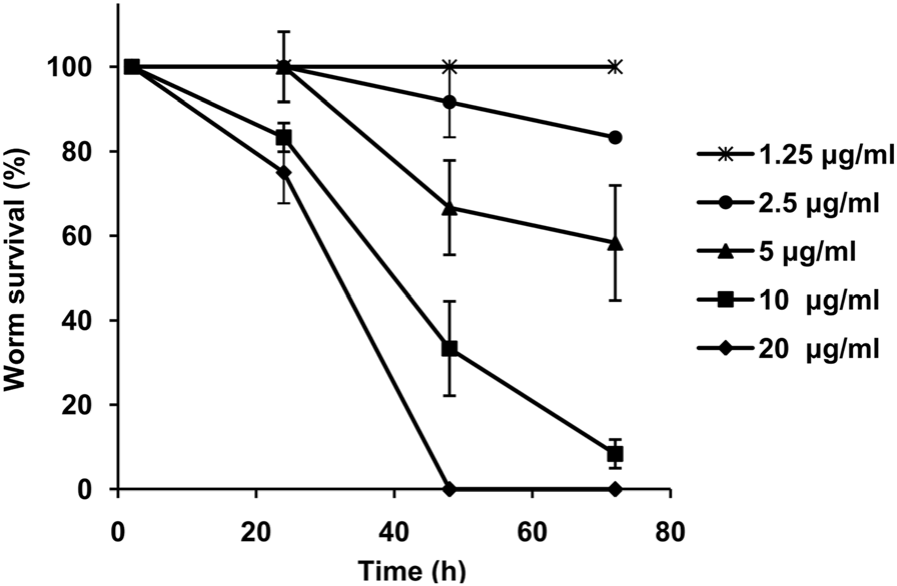
**Time-dependent survival rate of worms treated with different concentrations of compound AR9.** The concentrations of AR9 used were 1.25 μg/ml (asterisk), 2.5 μg/ml (circle), 5 μg/ml (triangle), 10 μg/ml (square) and 20 μg/ml (diamond).

The effects of the identified compounds on adult worms were observed by culturing the worms in medium containing different amounts of each compound, and death rates as well as morphological alterations were monitored. In the primary screening, two compounds, AR6 and AR9, exhibited good inhibition ability on cultured worms. AR6 resulted in 75% mortality after 72 h, but the effect was limited to 50 μg/ml. A similar effect was observed for AR9 with activity at a lower concentration; thus, it was selected for the secondary screening to confirm the efficacy of AR9 and further determine its LC50 value. Here, 10 μg/ml of AR9 resulted in 91.67% mortality over 72 h, and at 20 μg/ml, 100% mortality over 48 h was observed. In contrast, worms treated with equal volume of DMSO maintained a good activity throughout the whole experimental period (Figure 2). The 72 h-dependent LC50 value of AR9 calculated by SPSS 18.0 was 5.72 μg/ml (16.42 μM), with a 95% confidence interval of 4.37 μg/ml-7.91 μg/ml.

**Figure 3:**
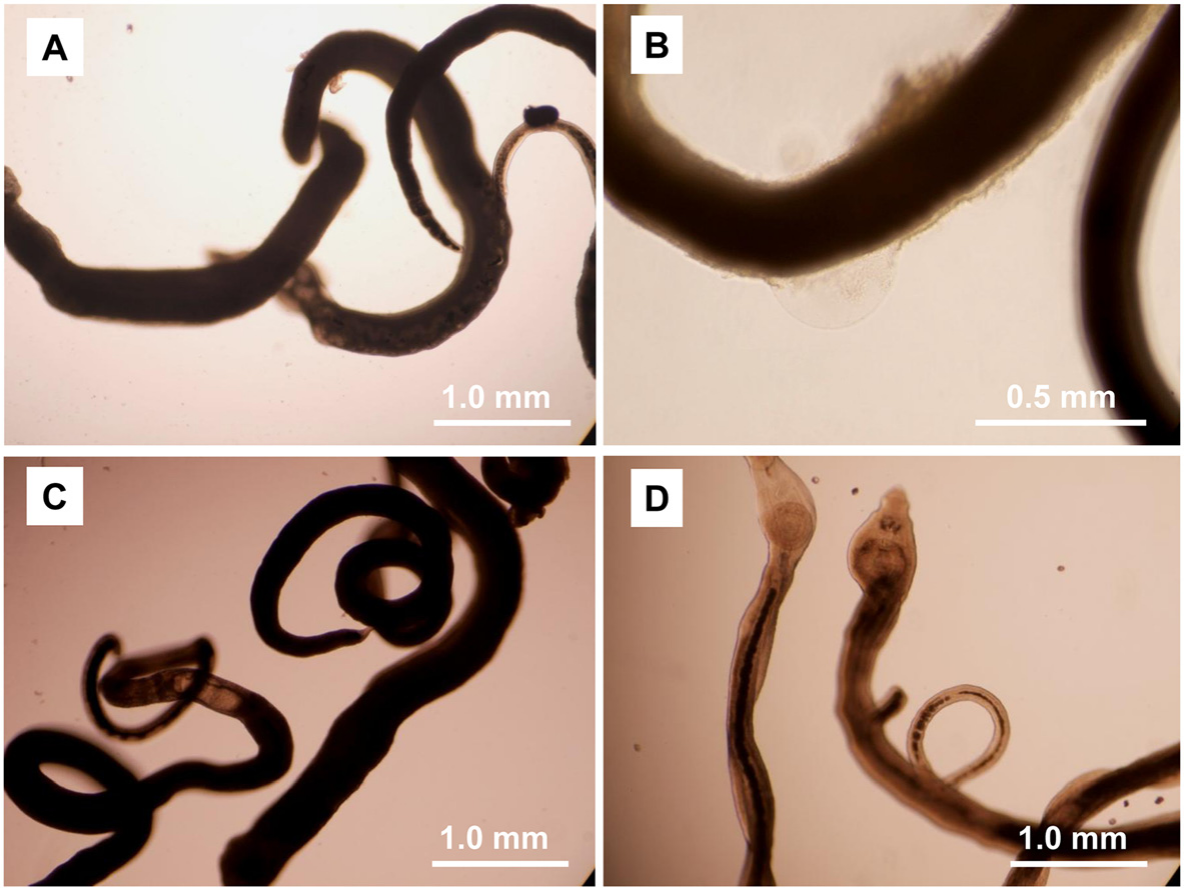
**Observation of worms exposed to compound AR9 under optical microscopy. A**. Worms treated with 5 μg/ml of AR9 for 48 h; **B**. treatment with 10 μg/ml of AR9 for 72 h; **C**. Positive control group, treatment with 5 μg/ml of PZQ for 48 h; **D**. Negative control, treatment with AR9 carrier only for 72 h.

Optical images revealed that worms treated with compound AR9 turned black, and obvious damage to the tegument was also observed (Figure 3A and 3B). As a positive control, PZQ induced local drastic contracture, which was consistent with previous studies [28] (Figure 3C). In contrast, these morphological alterations were not observed in the compound-carrier treated group (Figure 3D).

**Figure 4:**
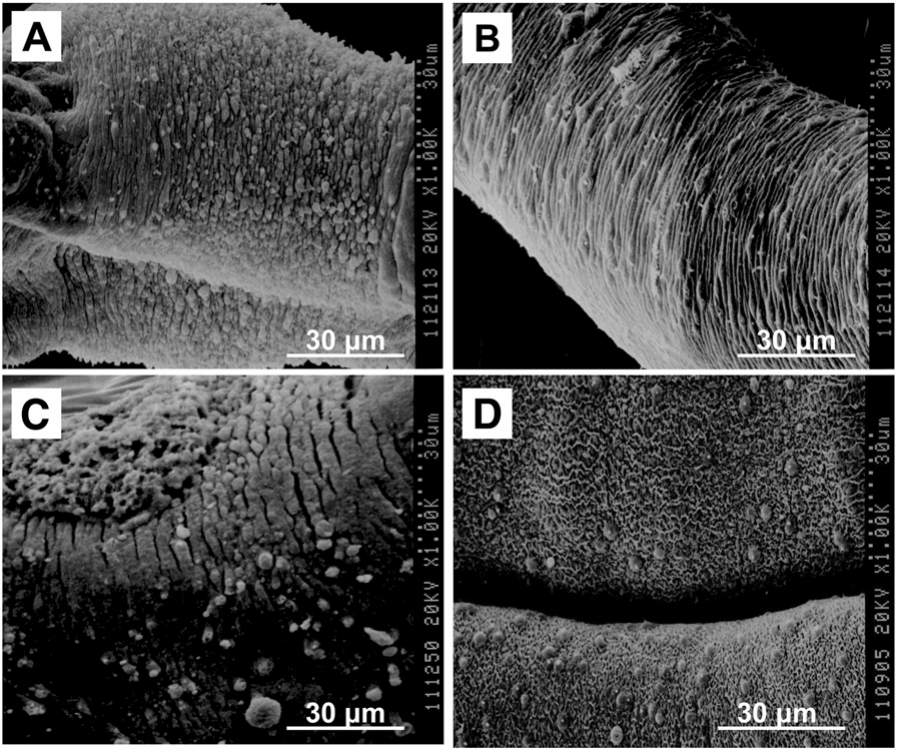
**SEM images of the tegument of adult worms. A** and **B** were worms treated with 10 μg/ml of AR9; **C**. Positive control group, treated with 10 μg/ml of PZQ; **D**. Normal control, paired worms treated with the AR9 carrier alone.

SEM images further confirmed the results of microscopy. Severe wrinkles and extensive small blebs were observed on the tegument layer of AR9-treated worms (Figure 4A and 4B), while PZQ treatment resulted in many ruptures of the tegument layer (Figure 4C)*.* In contrast, the surface of the schistosomes in the control group was very smooth and exhibited a dense network structure (Figure 4D).

### Compound AR9 target validation

**Figure 5:**
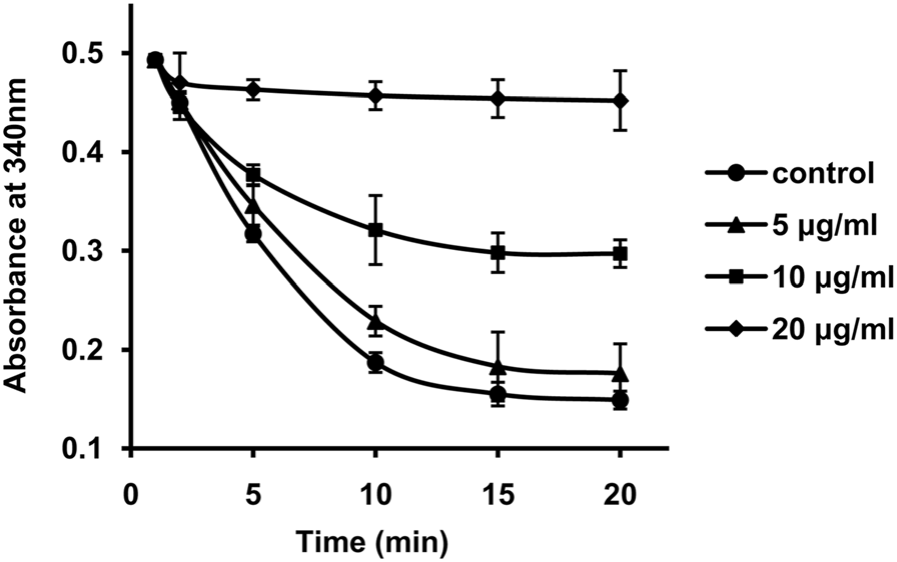
**The effect of compound AR9 on r *Sj*AR enzymatic activity.** The concentrations of AR9 were 5 μg/ml (triangle), 10 μg/ml (square) and 20 μg/ml (diamond). The AR9 blank was used as a control (circle). The r*Sj*AR concentration in each group was 0.15 μM. The assay was performed independently three times. The background was removed from the data shown here.

To verify that *Sj*AR is indeed the target of compound AR9, we assessed the effect of AR9 on r*Sj*AR enzymatic activity. A concentration-dependent inhibition of r*Sj*AR activity was observed. Compared with the compound-carrier-treated group (0.4% DMSO), AR9 at concentrations of 10 μg/ml and 20 μg/ml reduced r*Sj*AR activity by 42.31% and 88.46%, respectively (Figure 5). The half-maximal inhibitory concentration (IC50) calculated by SPSS 18.0 was 11.75 μg/ml (33.72 μM) in the first 10 min, with a 95% confidence interval of 8.87 μg/ml −12.57 μg/ml.

### Cytotoxicity assay

**Figure 6:**
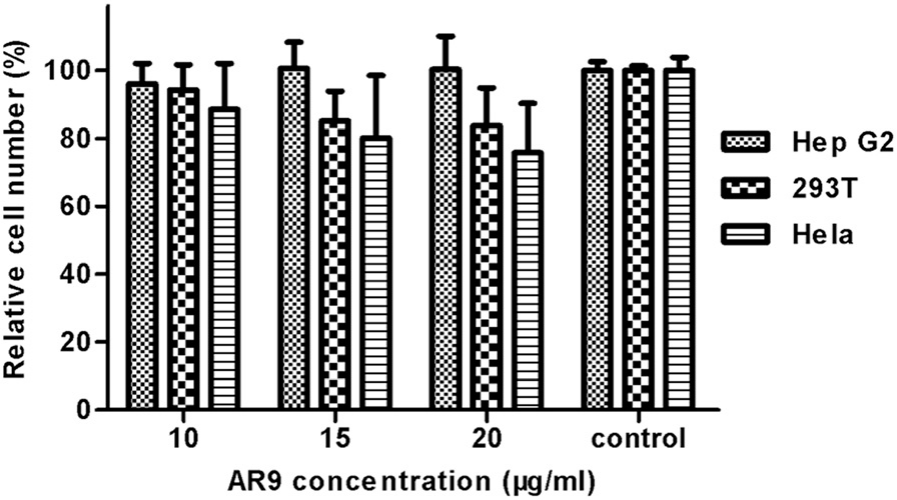
**The cytotoxicity of compound AR9 against host cells.** The cell activity of compound-carrier alone treated group was defined as 100%. The relative cell activity values of other experimental groups were calculated by comparing with the control group. The cytotoxicity was determined by Counting Kit-8 analysis, as described in the Methods section.

To assess the cytotoxicity of compound AR9, three different cell lines (liver carcinoma cells, Hep G2; kidney cells, 293T and breast cells, Hela) from *H. sapiens* were selected as experimental toxicity screens. For comparison, the activity of all the cells was not significantly affected by exposure to 20 μg/ml of AR9 for 72 h (especially in the Hep G2 cells, where almost no cytotoxicity was observed), while AR9 at 10 μg/ml led to over 90% worm mortality over the same time period (Figure 2 and Figure 6).

## Discussion

The antioxidant defense system plays a key role in the physiological functions of an organism [32]. For schistosomes, there have been many studies showing that this system protects the worms from host ROS attack; therefore, the associated enzymes are usually considered to be potential drug-discovery targets [11,33]. *S. japonicum* aldose reductase, an important enzyme involved in this system, was predicted as a potential drug target. In this study, we firstly obtained the *Sj*AR crystal structure through the X-ray diffraction method, on which a virtual screening and experimental validation strategy was applied to screen antischistosomal lead compounds. Finally, one compound, renamed as AR9, was determined to have effective antischistosomal activity but relatively low cytotoxicity towards host cells, which suggested that it has the potential as a lead compound from our selected candidate inhibitors for further drug development.

**Figure 7:**
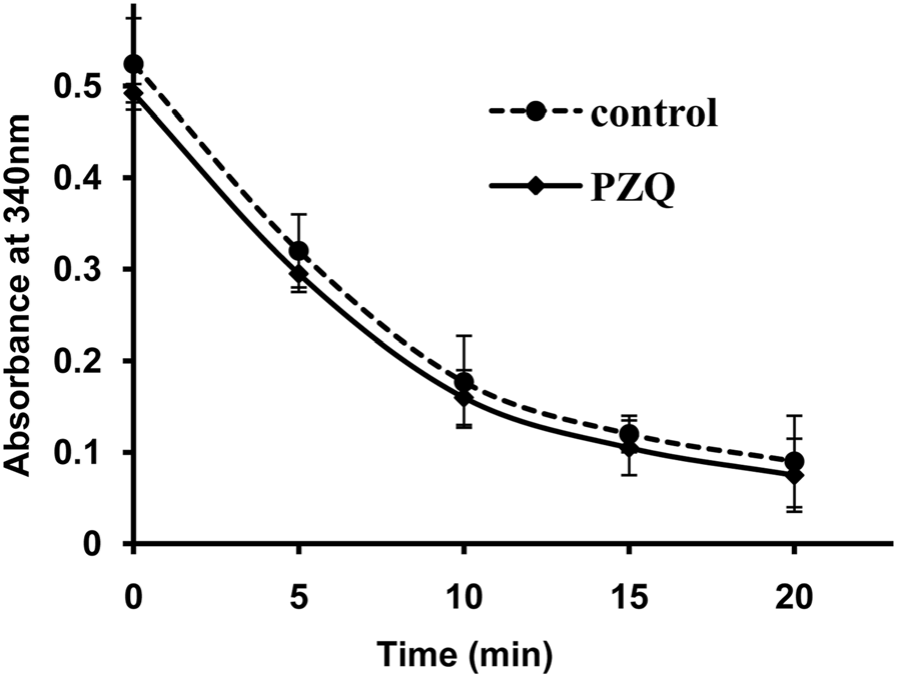
**The effect of PZQ on r *Sj*AR enzymatic activity.** The concentration of PZQ was 10 μg/ml (diamond), while PZQ blank group was used as control (circle). The r*Sj*AR concentration in each group was 0.15 μM.

Although no research has reported the *Sj*AR before, the *Hsa*AR has long been considered to be a potential target for therapies for diabetes and cancer [20,34], and a large number of inhibitors have been identified over the last 30 years. In this study, we also tested the inhibitory activity of two known *Hsa*AR inhibitors (epalrestat and quercetin) on cultured worms, but neither of them exhibited significant activity in *vitro* (data not shown). This may be because of their specificity for targeting *Hsa*AR protein or their failure to reach the target area in schistosomes. In contrast, the compound AR9 exhibited not only a strong inhibition of r*Sj*AR enzymatic activity (IC50 =11.75 μg/ml) but also significant inhibitory activity of cultured worms (LC50 =5.72 μg/ml). In this study, we also attempted to obtain the LC50 value of the current drug PZQ for comparison of the corresponding LC50 of AR9. It is of note that although the motor activity of worms exposed to different concentrations of PZQ (1.25 μg/ml-40 μg/ml) decreased significantly in a short time, however, the oral sucker and ventral sucker remained active for a relatively long period, and these worms couldn’t be judged as dead according to the death definition described in the Methods section, so we could not provide the accurate LC50 value of PZQ. We acknowledge that the efficacy of AR9 on schistosomes is not as good as PZQ, however, this study is still valuable. As shown in Figure 7, 10 μg/ml of PZQ exhibited no inhibition on r*Sj*AR enzymatic activity which indicates that compounds targeting *Sj*AR may have a different mechanism of action compared to PZQ. This result provides the foundation of combination therapy with AR9 and PZQ. Combination therapy is considered to be an effective approach to prevent the emergence of PZQ resistance. The effectiveness of artemisinin derivatives in combination with PZQ has been demonstrated [35]; however, because artemisinin derivatives are effective anti-malaria drugs, there is still concern about the induction of drug-resist malaria. The results from this study avoid that concern since these compounds have not been used as anti-malarial drugs.

In this study, we also tested the enzyme inhibition for all of the 10 small molecule compounds (Compound AR8 was not analyzed because of its low solubility) and we tried to find whether there was a positive correlation between enzyme inhibition and parasite growth inhibition. The results showed that, besides AR9, compounds AR1, AR3, AR5 and AR6 also exhibited a certain degree of inhibition on r*Sj*AR enzymatic activity (for AR1, AR3 and AR5, the IC50 values were less than 10 μg/ml, while for AR6, the IC50 value was greater than 20 μg/ml). However, none of these compounds showed significant inhibitory activity on the cultured worms, except that AR6 resulted in 75% mortality in 72 h at a concentration of 50 μg/ml. Therefore, no significant correlation was established between *Sj*AR inhibition and adult worm killing *in vitro*. The reason for this might be explained by other factors, such as compound molecular weight, solubility or their different pharmacokinetics *in vivo*, which could also affect the lack of correlation of the enzyme inhibition assay with activity on cultured worms.

The identified compound, AR9, has two linked anthraquinone scaffolds, and its name is bianthrone or dianthrone. Although anthraquinone scaffolds usually have multiple molecular targets which usually result in their promiscuity, there are indeed some cases that have successfully made the anthraquinone compound into drugs by introducing certain groups. Mitoxantrone, pixantrone and the anthracyclines, all of which are anthraquinone derivatives, have already been used as effective drugs for cancer treatment [36-38]. Additionally, rufigallol, another anthraquinone derivative, also exhibits significant toxic to the malaria parasite *Plasmodium falciparum*[39]. Therefore, although the current structure of AR9 seems unsuitable for a drug, improved derivatives could also be designed based on this structural model. Additionally, bianthrone is actually the major active ingredient of a widely used Chinese herbal medicine named *rheum palmatum*, which has been demonstrated to effectively inhibit bacteria, fungi and viruses [40,41]. These studies further support the speculation that AR9 has the potential to become a novel antischistosomal lead compound.

**Figure 8:**
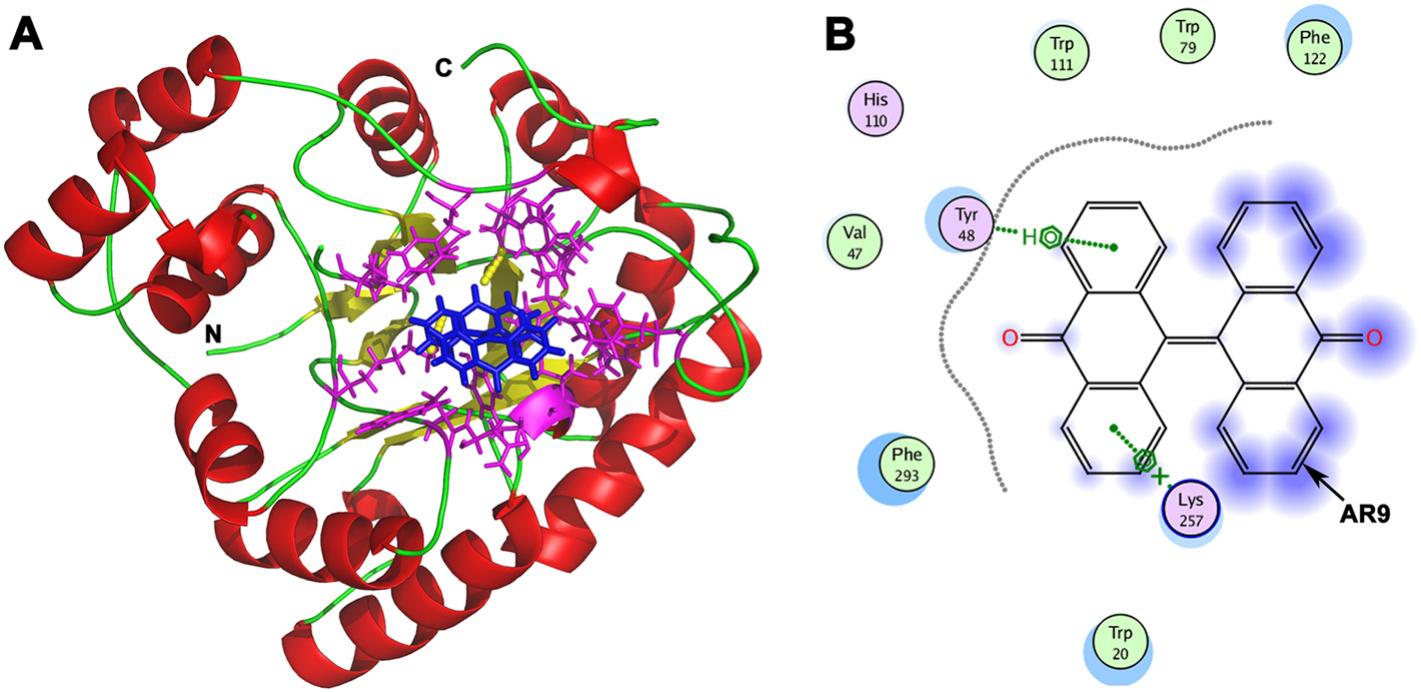
**The models of compound AR9 docked into the *Sj*AR structure. A**. The predicted overall structure of *Sj*AR bound with compound AR9. The structure of *Sj*AR is shown in a cartoon representation, with α-helices colored red, β-sheets colored yellow and loops colored green. Compound AR9, located in the interior of a hydrophobic pocket, is colored blue. The nearby residues surrounding AR9 are colored magenta and shown with a stick model. View is from the bottom of the (α/β) _8_ barrel. **B**. The interactions between compound AR9 and *Sj*AR. The arrow indicates compound AR9. Polar and non-polar amino acids are colored magenta and green, respectively. Blue background on AR9 structural model indicates the ligand exposure area. The figure is generated with software Molecular Operating Environment 2011 (MOE).

Molecular docking analysis showed that AR9 located to the interior of the pocket composed of the residues Val^47^, Tyr^48^, Trp^79^, His^110^, Trp^111^, Phe^122^, Lys^257^ and Phe^293^. AR9 can bind with the residues Tyr^48^ and Lys^257^ through the established hydrogen bonds (Figure 8). It is noteworthy that although AR9 has a two-fold symmetry in its scaffold, only one of the anthraquinone moieties interacts with the *Sj*AR protein, so the second moiety seems redundant. However, when we removed the other half of the AR9 structure and performed the docking process again, the resulting prototype anthraquinone structure showed poor binding ability with *Sj*AR protein. A significantly difference was that the previously existing hydrogen bonds between AR9 and *Sj*AR protein residues (Tyr^48^ and Lys^257^) were not established as expected, and meanwhile, no other residues newly-participated in the combination with the prototype anthraquinone. The result might be explained that the second moiety changing the distribution of the electron cloud of the binding regions, thereby facilitating the binding of the first moiety. However, the true binding model between a small molecule and its target protein can only be obtained by analyzing the *Sj*AR-AR9 complex structure.

In this study, we have attempted to obtain the crystal structure of *Sj*AR complexed with compound AR9 through co-crystallization. However, this was very difficult to achieve because of the relatively lower solubility of AR9. AR9 is soluble in DMSO, but less soluble in water. When the concentration of AR9 in water was higher than 50 μg/ml, some precipitation would occur. Meanwhile, the optimized *Sj*AR protein crystallization concentration is 12 mg/ml (approximately 0.31 mM), so even assuming that one *Sj*AR protein molecule only binds to one compound molecule, the minimum concentration of AR9 should be 119 μg/ml. However, large amounts of precipitation have already occurred at that concentration. An alternative strategy is to introduce a polar group in the AR9 structure (ensuring that this change does not significantly affect its antischistosomal activity) to increase its solubility, and then attempt co-crystallization.

The majority of previous studies have focused either on the screening and designing of inhibitors of a known drug target or on the analysis of antischistosomal activity of potential drugs [12,42,43], while the work presented here bridges the gap between virtual screening and experimental validation, providing an effective and economical strategy to discover antischistosomal lead compounds. More work, such as in *vivo* experiments, the design of derivatives and optimization of complex crystallization conditions are still needed in further studies.

## Conclusions

The work presented here developed an effective and economical strategy, which integrates virtual screening and experimental validation for the development of new anti-parasitic drugs. In this study, we firstly resolved the *Sj*AR structure and identified one compound, bianthrone, which may become a new potential lead compound for developing novel antischistosomal drugs based on this structural model.

## Competing interests

The authors declare that they have no competing interests.

## Authors’ contributions

WH, ZY and XNW conceived and designed the study. JL, DHD, JDC, JPW and SQW performed the experiments, analyzed the data and drafted the manuscript. WH and DHD revised and finalized the manuscript. All of the authors read and approved the final version of the manuscript.
